# Perceived stress in the time of COVID-19: the association with brooding and COVID-related rumination in adults with and without migraine

**DOI:** 10.1186/s40359-021-00549-y

**Published:** 2021-04-30

**Authors:** Lilla Nóra Kovács, Dániel Baksa, Dóra Dobos, Nóra Eszlári, Kinga Gecse, Natália Kocsel, Gabriella Juhász, Gyöngyi Kökönyei

**Affiliations:** 1 Doctoral School of Psychology, ELTE, Eötvös Loránd University, Budapest, Hungary; 2 SE-NAP2 Genetic Brain Imaging Migraine Research Group, Hungarian Brain Research Program, Semmelweis University, Budapest, Hungary; 3 Institute of Psychology, ELTE, Eötvös Loránd University, Budapest, Hungary; 4 Department of Pharmacodynamics, Faculty of Pharmacy, Semmelweis University, Budapest, Hungary; 5 NAP-2-SE New Antidepressant Target Research Group, Hungarian Brain Research Program, Semmelweis University, Budapest, Hungary; 6 MTA-SE Neuropsychopharmacology and Neurochemistry Research Group, Hungarian Academy of Sciences, Semmelweis University, Budapest, Hungary

**Keywords:** COVID-19, Perceived stress, Migraine, Depressive rumination, Rumination, Brooding, COVID-related rumination

## Abstract

**Background:**

The main goal of this research was to explore whether migraineurs had a higher level of perceived stress than healthy controls during the times of the coronavirus and related restrictive measures, and to examine the relationship between different subtypes of rumination and perceived stress in these groups. We measured two facets of depressive rumination, brooding and reflection, along with rumination about the current COVID-19 situation to see whether these different subtypes of rumination explained perceived stress among migraineurs and healthy controls.

**Methods:**

Healthy adults (n = 64) and migraine patients (n = 73) filled out self-report questionnaires online. A multiple linear regression model was used to test whether depressive rumination (i.e. brooding and reflection) and COVID-related rumination explained perceived stress among adults with and without migraine during the times of COVID-19, after controlling for gender, age, migraine/control group status and migraine disability.

**Results:**

Although we did not find any difference in the level of perceived stress among migraineurs and the control group, perceived stress was more strongly associated with brooding as well as COVID-related rumination among migraineurs than healthy controls. COVID-related rumination and brooding (but not reflection) explained the level of perceived stress after controlling for gender, age, migraine/control group status and migraine disability.

**Conclusions:**

The similar degree of perceived stress among migraineurs and the control group may imply that there is great variation in the personal experience of people regarding the pandemic, that may be determined by numerous other factors. Our results demonstrate that ruminating about the pandemic and related difficulties, as well as brooding (but not reflection) appear to be associated with higher level of perceived stress during the times of the coronavirus. This association was slightly stronger among migraineurs, hinting at the increased vulnerability of this patient group in stressful situations like the COVID-19 pandemic. Our results also suggest that ruminating about the pandemic and its consequences is weakly associated with trait-level depressive rumination, thus may be more contingent on specific factors.

## Introduction

Besides directly threatening people’s health, the COVID-19 pandemic has severe consequences for everyday life via bringing about financial insecurity, social distancing and restrictive measures [1]. Thus, the situation since March 2020 until now can be considered a severe stressor affecting most of the population. COVID-19 and related restrictions may cause significant changes in people’s psychological symptoms [2, 3], provoking a secondary mental health crisis [4].

Stress has long been a significant concept of study in health science since has been vastly associated with different health outcomes [5], however, there is great variability in its definition [6]. In stress-related research, stress can be broadly conceptualized in three different ways. The environmental approach focuses primarily on the outer stressor, the psychological approach targets the person’s subjective evaluation of a stressful situation and their psychological response to it, while the biological approach mainly investigates the physiological responses given to a stressor [7]. In the current study we aimed to conceptualize stress according to the psychological approach, i.e. we were investigating to what extent people consider their life conditions frustrating or overwhelming [8], as this is a crucial aspect of withstanding the challenges provoked by COVID-19 [9]. Within this approach, our conceptualization is in accordance with Lazarus and Folkman’s definition of stress as the product of the discrepancy between perceived external challenges and the individual's subjective intra- and interpersonal capacity to live up to those challenges [10].

Empirical evidence from stress-related research underlines that the cognitive-emotional response given to stressful situations may play a more important part in adaptation than the stressor itself [11, 12]. Rumination, i.e. the continuous unproductive dwelling on a negative event [13] can be considered as a maladaptive stress response, as it may exacerbate the importance of the perceived stressor and may lead to serious negative psychological [14] and physiological [15] outcomes. By mentally representing a stressor that may not actually be present or by elevating its perceived importance, rumination can trigger a fight-or-flight stress response with physiological consequences, such as elevated heart rate and cortisol level [16]. By constantly recalling the stressor even without its presence, it may prolong stress exposure and negatively impact mental and physical well-being [15]. Although people generally think that constantly dwelling on stressful situations facilitates problem solving and choose this coping strategy on purpose, research suggests that it is hardly productive, and should rather be considered a maladaptive reaction to stress [14]. Therefore, exploring rumination in difficult, stress-provoking times like the COVID-19 pandemic is of utmost importance to understand stress response and psychological well-being.

Rumination can be categorized in various subtypes based on the content of ruminative thinking. According to its most widespread conceptualization, the Response Style Theory, rumination can be described as recurrently thinking about the concomitants of one’s own negative mood and depressive symptoms, i.e. depressive rumination [13]. Depressive rumination can be categorized as brooding and reflection, where brooding is considered a maladaptive, self-criticizing aspect of recurrent thinking about emotionally relevant life events, while reflection is a more constructive thinking style that may foster problem solving [17]. Brooding has been repeatedly associated with depressive symptoms [18, 19], anxiety [20] and prolonged stress reaction [16]. Additionally, the current situation can be considered a serious stressor that may evoke ruminative thoughts regarding the pandemic and its possible consequences such as people’s health, occupation, personal relationships etc. [21]. In support, recent findings suggest that COVID-related ruminative thoughts may elevate the perceived importance of the stressor and enhance perceived stress, similarly to depressive rumination [22]. Trait-level depressive rumination has been associated with elevated stress-related physical, behavioral and psychological symptoms in the presence of COVID-related stressors [21], while COVID-specific rumination was found to be associated with distress, fatigue and depressive symptoms [23]. However, the relationship between different subtypes of rumination and their specific contributions to perceived stress have not been reported elsewhere. Thus, investigating the associations between psychological distress and both depressive and COVID-related rumination is important, and may be especially relevant among patients with diseases related to stress [24] and rumination [25], such as migraine.

Psychological stress, i.e. the perceived disparity between demands and one’s own capacities [10], has been robustly reported as the most frequent factor to provoke migraine attacks [26–28], as well as to prolong their duration [29]. Migraine is a common and debilitating headache disorder affecting more than one billion people worldwide [30]. However, there are still many uncertainties about the exact causes for the activation of attacks, as it probably comprises a complex interplay of genetic and environmental factors [31, 32]. As a painful disease, migraine attenuates the quality of life [33] and has been associated with elevated levels of anxiety and depressed mood [34], that are well-known concomitants of rumination [35]. A recent fMRI study found elevated brain activation to faces expressing fear among migraine patients compared to healthy controls (HCs) in areas associated with the attentional network, suggesting that these patients may be more vigilant to potentially threatening stimuli [36]. Hypersensitivity to potentially threatening stimuli and ruminating in response to negative events—frequently observed among migraine patients [37]—may elevate the perceived importance of the stressor and hamper adaptation [35, 38]. Correspondingly, a study found that migraine patients reported more rumination and higher levels of anxiety and depressed mood than HCs, and the connection between migraine and symptoms of anxiety and depression was mediated by brooding in two independent European samples [25]. Thus, it appears that personal characteristics in emotion regulation strategies such as rumination may also contribute to the level of psychological well-being of migraine patients [25]. In addition, anticipating and experiencing a migraine attack is a great source of stress itself, resulting in a negative spiral [26]. Taken together, patients with migraine may be more prone to magnify stressful events due to their hypersensitivity to threatening stimuli and their elevated tendency to ruminate compared to HCs, further elevating their level of perceived stress. Therefore, this group may be especially vulnerable to develop stress-related symptoms during the current situation caused by the COVID-19 pandemic and related restrictive measures.

In the current study we aimed to explore whether the level of perceived stress was higher among migraineurs than HCs, and whether migraine status and rumination predicted elevated perceived stress, after controlling for gender, age, and disability caused by headache. We aimed to distinguish rumination about COVID-19 from depressive rumination, as the former may be an acute, specific response to the current situation that probably characterizes most of the population nowadays [39], whereas the latter is considered a more stable personality trait that is not distinctive to the current situation (although may be enhanced by it). To date, the association between COVID-related rumination and depressive rumination—i.e. whether people who tend to dwell on their depressed mood may or may not engage in rumination specific to COVID-19—has not been reported, thus we also aimed to explore their relationship in this study. Additionally, as brooding is considered a maladaptive aspect of rumination, whereas reflection may be more constructive [17], we expected brooding to show a stronger relationship with perceived stress than reflection. Furthermore, we assumed that perceived stress will be the highest among migraineurs who display high levels of rumination. In other words, we expected an interaction between migraine status and brooding—the maladaptive facet of depressive rumination—in predicting the perceived level of stress. In the same vein, we also aimed to explore whether there was an interaction between migraine status and COVID-related rumination, which, according to our knowledge, has not been studied elsewhere.

## Methods

### Sample and procedure

Data analyzed in the current study was collected in May–June 2020. We contacted 311 people who had participated in previous studies between 2014 and 2019 and agreed to be approached for future research. Inclusion criteria for these previous studies included aged between 18 and 50 years, and no history of severe somatic, neurological or psychological problems—except migraine—or psychotropic medication. In order to verify these criteria, potential subjects first had to undergo an interview where a trained research assistant administered the Mini International Neuropsychiatric Interview [40] to screen for potential psychiatric disorders and explored the medical history of the participant. If found eligible (for these previous studies), participants had to attend a medical examination by a headache specialist, who established the diagnosis of episodic migraine without aura based on the International Classification of Headache Disorders-III criteria (ICHD-III, beta version; Headache Classification Committee of the International Headache Society (IHS, 2013).

Power analysis for the current study was conducted using G*Power software [41]. An estimated minimum sample size in a linear regression containing eight predictors, with an expected medium effect size of 0.15 [42] necessary to gain 0.80 power was 109. We sent the link of the study to 311 potential respondents in e-mail. Participation was anonymous and voluntary, informed consent was acquired. 73 patients with episodic migraine without aura and 64 HCs filled out the questionnaires. Four people reported that they have been in quarantine designated by the epidemiological authority, and only one participant reported to have been tested positive to COVID-19. We considered these factors as high-level stressors that may influence the perceived level of stress and rumination regarding COVID-19 substantially, thus we excluded the affected participants from the analyses. No participants reported to have lost a relative or close acquaintance due to COVID-19 (otherwise, they would have been excluded for the same reason). The final sample comprised of 132 participants. The sample was predominantly female (73.5%; n = 97), and highly educated: 21.2% had a high school diploma, 74.8% had a university degree. The minimum age was 20, the maximum 50 years (M = 30.76; SD = 7.10). The original study, as well as the current data acquisition was approved by the Scientific and Research Ethics Committee of the Medical Research Council (Hungary) and is in accordance with the Declaration of Helsinki.

### Measures

Demographic data (gender, age, education), and potential confounding factors related to the pandemic were assessed. We asked participants whether they had been obliged to stay in quarantine by the epidemiological authority or chose to stay in quarantine voluntarily since the outburst of the COVID-19 in Hungary (March, 2020), whether they or their close family members tested positive to COVID-19, and whether they lost a relative or close acquaintance due to COVID-19.

The 10-item Ruminative Response Scale (RRS; 43) was used to measure depressive rumination, where respondents are instructed to evaluate their repetitive thinking style when feeling sad or depressed. The RRS contains two subscales, brooding and reflection, each measured by 5 items rated on a four-point Likert scale from 1 (never) to 4 (always). Brooding is considered a maladaptive, often self-blaming aspect of repetitive thinking about stressful life event. Reflection, on the other hand, is a more constructive way of rumination that may facilitate problem solving [17]. The brooding and reflection subscales of the Hungarian RRS have shown good internal consistency in a previous study (Cronbach α = 0.71 and Cronbach α = 0.73, respectively) [25], as well as in the current sample (Cronbach α = 0.71 for brooding and Cronbach α = 0.70 for reflection).

The four-item Perceived Stress Scale (PSS-4; 8) was used to measure how participants appraised their own levels of stress in their lives during the past 3 months. We defined this time period because it corresponded to the appearance of the COVID-19 pandemic in Hungary, hence it covered a potentially stressful period for most people due to the threat of the virus, restrictive measures and social distancing. Items are rated on a five-point Likert scale ranging from 0 to 4, two of which are positively stated and reversed. The PSS-4 have demonstrated good psychometric properties in various studies [44, 45]. The Hungarian PSS-4 demonstrated good internal consistency in a previous study (Cronbach α = 0.79) [46], as well as in this sample (Cronbach α = 0.85).

COVID-related Rumination Scale (CRS) consisted of four items retrieved from the Post-event processing questionnaire (PEPQ; 47) that measures repetitive thoughts after a stressful social situation. The instruction and the wording of the items were tailored in order to focus on the content of repetitive thinking regarding COVID-19. Participants were instructed to think about the current COVID-19 situation and related events (e.g. reports on new cases and mortality) and restrictive measures and indicate to what extent have they experienced these processes. Modifications and translation to Hungarian were carried out by Gy.K., N.K. & L.N.K. We aimed to capture the intrusive nature of repetitive thoughts *(1. My memories and thoughts about the event keep coming into my head even when I do not wish to think about it; 2. Thoughts about the event interfere with my concentration.)* and the amplification of the perceived stressor *(3. When I think about coronavirus over and over again, my feelings about the event get stronger/more negative.).* In addition, Item 7 of the PEPQ (Did you try to resist thinking about the event?) was altered more exhaustively, as the verb ‘resist’ already implies repetitive recurrent thoughts about the event—besides the difficulty to stop these thoughts—which may not apply to everyone. Thus, we aimed to separate these two assumptions by rephrasing it as “*4. If I start thinking about these things, I find it difficult to stop.*” to capture the difficulty to control repetitive thoughts. Cronbach α of the CRS was 0.84 in the current sample.

The Migraine Disability Assessment (MIDAS; 48) questionnaire was used to measure the burden caused by headache. As migraine attacks and everyday activities missed due to headache are great sources of stress by themselves, we aimed to control for the number of days with debilitating headache in the regression model. Scores of the first five items of the scale was summed for each participant to capture headache-related disability (e.g. missed days and/or reduced productivity in work/school, household and social activity due to headache) in the last three months. Because of the COVID-19 situation, an additional instruction was added to the first item assessing missed work/school days: “*If you are at home because of the pandemic, how many days did you skip work or school due to headache in the past 3 months?*”. Similarly, the fifth item (missed days in family, social and leisure activity) was completed with the following sentence: “*If you are at home because of the pandemic, also count online or home family, social or leisure activities.*” We assessed the MIDAS among HCs as well and asked them to answer these questions regarding their headaches in general (if they had any).

### Statistical analyses

Statistical analyses were performed with IBM SPSS Statistics for Windows, version 25.0 (IBM Corp., Armonk, N.Y., USA). Descriptive statistical analyses and reliability testing of the assessed measures were carried out first. We tested whether there was significant difference in gender and age between the migraine and the HC group. In order to address the effects of potential confounding factors, possible significant differences in the level of perceived stress were examined between those who stayed in quarantine voluntarily and those who did not; and also between participants who had a family member (or members) infected with COVID-19 and those who did not have such relatives.

Correlations of the assessed scales were calculated for the total sample and for the migraine and HC groups separately. Furthermore, we estimated whether the difference in the correlational coefficients between the two groups was significant as suggested by Eid et al. [49]. Then, we conducted a multiple linear regression on the obtained data to test whether depressive rumination—especially brooding—and rumination specific to COVID-19 (measured by the CRS) explained perceived stress during the times of the coronavirus, after controlling for gender, age, headache status (i.e. migraine/HC group), disability due to headache (i.e. the MIDAS score). We entered our variables to the model in three blocks starting with gender, age, headache status and disability due to headache, followed by the CRS in the second step, and the brooding and reflection subscales of the RRS in the third step. In the fourth step, we also aimed to test whether there was an interaction between brooding and headache status regarding perceived stress, for which we centered the brooding variable. We performed post-hoc tests to check for model assumptions, i.e. homoscedasticity, multicollinearity and the normal distribution of residuals.

## Results

Descriptive statistics of age and the assessed measures are shown in Table 1 for the total sample, as well as for the migraine and the control group separately. Participants in the migraine group were slightly older and—as expected—showed higher level of migraine-related disability than HCs, but no other significant differences were found between the two groups in brooding, reflection, COVID-related rumination and perceived stress. We performed Mann–Whitney U tests due to the non-normality of the variables. Besides age, gender distribution (χ^2^ = 14.27, *p* < 0.001) of the participants showed significant difference between the two groups: there were 9 males and 61 females in the migraine group, whereas there were 26 males and 36 females in the HC group. This is not surprising given that migraine is much more common among women [50]. Thus, we controlled for gender and age in the regression model.

**Table 1:** Descriptive statistics of age, assessed measures, and group differences between participants with and without migraine

Scales	Total sample Mean (SD) n = 132	Migraine group Mean (SD) n = 70	HC group Mean (SD) n = 62	Mann–Whitney U	*p*
Age	30.76 (7.10)	31.86 (7.28)	29.48 (6.73)	2597	.05
MIDAS	5.81 (9.18)	9.91 (10.70)	1.18 (3.23)	3803	< .001
RRS brooding	10.13 (2.90)	10.33 (2.99)	9.90 (2.81)	2274	.53
RRS reflection	11.79 (2.93)	11.78 (3.08)	11.81 (2.77)	2118	.92
CRS	6.20 (2.71)	6.56 (3.01)	5.81 (2.28)	2402	.28
PSS-4	5.64 (2.99)	5.84 (3.08)	5.41 (2.90)	2350	.41

*MIDAS* Migraine Disability Assessment, *RRS* Ruminative Response Scale, *CRS* COVID-related Rumination Scale, *PSS-4* Perceived Stress Scale, *SD* standard deviation

We estimated the effect of potential confounding factors, i.e. stayed in voluntary quarantine (n = 47) or not (n = 85), family member infected with COVID-19 (n = 11) or not (n = 121) by examining group differences regarding perceived stress with Mann–Whitney *U* tests. None of these group differences were significant (Mann–Whitney *U* = 1897, *p* = 0.632; Mann–Whitney *U* = 650, *p* = 0.898, respectively), thus we did not include them in the regression model as control variables.

We performed Spearman correlations of the assessed measures for the total sample and for the migraine and HC group separately. Non-parametric correlations were applied due to the non-normality of the variables. The results are summarized in Table 2.

**Table 2:** Spearman correlations of the assessed measures for the total sample and for the migraine and control group separately

	Total sample n = 132				Migraine group n = 70				Control group n = 62			
	RRS r	CRS	PSS	MIDAS	RRS r	CRS	PSS	MIDAS	RRS r	CRS	PSS	MIDAS
RRS b	.27**	.24**	.49**	− .04	.28*	.30*	.58**	− .14	.27*	.13	.41**	− .24
RRS r		− .01	.08	.05		− .07	.05	.04		.06	.12	.02
CRS			.32**	.06			.44**	.03			.12	− .23
PSS				.13				− .02				.21

*RRS b.* Ruminative Response Scale brooding subscale, *RRS r* Ruminative Response Scale reflection subscale, *CRS* COVID-related Rumination Scale, *PSS* Perceived Stress Scale, *MIDAS* Migraine Disability Assessment

*p < 0.05; **p < 0.01

As Table 2 demonstrates, brooding, COVID-related rumination and perceived stress were significantly correlated in the total sample. However, when examined separately, COVID-related rumination correlated with perceived stress and brooding only in the migraine group. Where correlation coefficients differed substantially between the two groups, we calculated whether these differences were significant following the method suggested by Eid et al. [49]. We found a tendency-level difference in the correlation coefficients in case of brooding and perceived stress (Z = 1.27, *p* = 0.10), and significant difference in case of COVID-related rumination and perceived stress (Z = 1.97, *p* = 0.02), where the association was stronger in the migraine group (r = 0.44, *p* < 0.01) than among HCs (r = 0.12, *p* = 0.37). Since this difference may indicate that the association between COVID-related rumination and perceived stress is stronger among migraineurs than HCs, we also tested whether there is an interaction between migraine status and COVID-specific rumination in the regression model. We consider this an explorative step based on the group differences that emerged from the correlational analyses, as the available data in this subject is still scarce. Multiple linear regression was used to test whether rumination specific to COVID-19 (as measured by the CRS) and depressive rumination (i.e. brooding and reflection, as measured by the corresponding subscales of the RRS) explained perceived stress (as measured by the PSS) during the times of the coronavirus, after controlling for gender, age, headache status (i.e. migraine/HC group) and disability caused by headache (as measured by the MIDAS). We entered our variables to the model stepwise starting with gender, age, migraine/HC group status and the MIDAS score, followed by the CRS in the second, the brooding and reflection scales in the third step, and the interaction terms of brooding-headache status and COVID-related rumination-headache status in the fourth step. CRS and brooding were significant predictors of perceived stress, where more rumination predicted higher levels of perceived stress, after controlling for gender, age and headache status. We also aimed to test whether there was an interaction between brooding and headache status regarding perceived stress, however, we did not find significant interaction, and accordingly the change in R^2^ was not significant. The total explained variance of the regression model was 31.3% (*R*^2^ = 0.313; *df* = 130). Then, we included the interaction between COVID-related rumination and headache status in our model instead, however, this interaction was not significant either (β = 0.052; *p* = 0.693), and only resulted in marginal change (F(1, 122) = 0.16, *p* = 0.69) in the total explained variance (R^2^ = 0.308; df = 130). Model assumptions, i.e. the normal distribution of the standardized residual, homoscedasticity and multicollinearity were fulfilled. The model is presented in Table 3.

**Table 3:** Multiple linear regression model with subtypes of rumination explaining perceived stress, after controlling for gender, age, headache status and headache disability

Model	Predictors	β	*p*	*R*^2^
1	Gender	− .029	.762	.012
	Age	− .067	.458	
	Migraine/HC	− .080	.459	
	MIDAS	.034	.737	
2	Gender	− .091	.315	.138
	Age	− .036	.668	
	Migraine/HC	− 0.45	.656	
	MIDAS	.032	.734	
	CRS	.364	< .001	
3	Gender	− .049	.548	.308
	Age	.004	.957	
	Migraine/HC	.034	.711	
	MIDAS	.117	.182	
	CRS	.260	.001	
	RRS brooding	.448	< .001	
	RRS reflection	− .050	.536	
4	Gender	− .039	.644	.313
	Age	.009	.909	
	Migraine/HC	.042	.650	
	MIDAS	.125	.155	
	CRS	.255	.002	
	RRS brooding	.373	.002	
	RRS reflection	− .053	.512	
	RRS brooding * Migraine/HC	.105	.365	

*n* = 132

*RRS* Ruminative Response Scale, *CRS* COVID-related Rumination Scale, *PSS* Perceived Stress Scale, *MIDAS* Migraine Disability Assessment

## Discussion

Ruminating on current problems and adverse events may be a risk factor for the onset and exacerbation of various psychiatric and somatic symptoms [14, 15], therefore exploring repetitive negative thinking styles is particularly important in the current situation among the whole population, as well as in vulnerable subgroups such as migraine patients, who may be especially sensitive to stressful life events [24]. At present, not much data is available about the effect of COVID-19 on migraineurs’ well-being, and the available information is ambiguous. According to our knowledge, this is the first study that examines the associations between perceived stress and rumination during the COVID-19 pandemic among migraineurs. In the current study, we found no difference between migraineurs and the control group in the degree of perceived stress, brooding, or COVID-related rumination. Perceived stress was correlated more strongly with brooding as well as COVID-related rumination among migraine patients than HCs. The level of perceived stress was explained by both rumination subtypes, COVID-related rumination and brooding, but not reflection, after controlling for gender, age, migraine/control group status and migraine impairment.

Owing to their hypersensitivity to threatening stimuli and their increased propensity to ruminate relative to HCs, migraine patients may be more vulnerable to amplifying stressful experience, further increasing their level of perceived stress. This group may therefore be particularly vulnerable to the development of stress-related symptoms during the current situation triggered by the COVID-19 pandemic. Therefore, we explored whether the level of perceived stress was higher among migraine patients and HCs, however, we did not find significant difference in the level of perceived stress among the two groups. Also, despite stress being the primary trigger of migraine attacks and the attacks themselves being significant stressors [45], perceived stress did not show any association with migraine-related disability in our sample. One possible explanation is that many migraineurs experience more frequent migraine attacks not during the times of heightened level of stress, but when stress decreases [51]. Furthermore, some clinicians report that migraine patients seek treatment more frequently since the beginning of the pandemic [52] and that the majority of patients report more frequent and more severe attacks [53], while others observed a decrease in migraine attack frequency during this period [54]. A novel study showed a decrease in migraine frequency as the number of days spent home increased during COVID-19 [55], suggesting that the relief of staying at home may exceed the stress and anxiety related to the pandemic for some people, and underlying the substantial interindividual differences in the subjective experience of the pandemic and related restrictions. Moreover, our participants suffered from episodic migraine, and a recent study has shown that after controlling for education, depression and anxiety, perceived stress was higher among patients with chronic migraine, but not among patients with episodic migraine compared to HCs [62].

Migraineurs and HCs did not differ in the level of brooding, reflection, COVID-related rumination and perceived stress either, however, we found certain differences regarding the association of these measures within the two groups. Brooding was associated with perceived stress in both groups; however, a stronger correlation was found among migraineurs demonstrating a tendency-level difference between the two groups. Furthermore, COVID-related rumination and brooding were uncorrelated among HCs and only showed moderate association in the migraine group, indicating that people may find themselves dwelling on the pandemic and its concomitants regardless of their general tendency of brooding. It appears more plausible that COVID-related ruminative thoughts are triggered by specific problems, such as losing one’s job, financial difficulties, social isolation, difficulties related to homeschooling, worrying about one’s own health or the health of elderly relatives etc., and may be less contingent on one’s general tendency to dwell on negative events.

We also investigated whether migraine status and higher depressive rumination—especially its maladaptive form, brooding—explained elevated perceived stress, after controlling for potential confounds, namely gender, age, headache status and migraine disability. Also, we considered it important to examine the relationship between COVID-related rumination and perceived stress, as the whole population of the world is exposed to the pandemic as a constant stressor and faces its consequences in everyday life, and rumination may enhance the perceived threat of these difficult times [51]. Our results revealed that both COVID-related rumination and brooding were significant predictors of perceived stress in the total sample, and brooding significantly contributed to the explained variance of perceived stress after controlling for COVID-related rumination, implying that this self-focused, self-blaming subtype of ruminative thinking may be an important risk factor in the current situation, and not only among migraineurs. However, we could not find an interaction neither between headache status and brooding, nor between headache status and COVID-related rumination.

Taken together, although neither groups were characterized by more rumination or higher levels of stress, the differences in the correlations indicate that rumination, especially about COVID-19, may be more strongly associated with perceived stress among migraineurs than HCs. This result may hint at the vulnerability of this group to stressful situations like the current coronavirus pandemic. However, we need to interpret these results carefully, as it is only supported by the correlational analysis, while we did not find significant interaction neither between brooding and headache status, nor between COVID-related rumination and headache status in the regression model.

Our study has certain limitations. Most importantly, our cross-sectional study design does not allow us to infer causation, and it is important to keep in mind that the relationship between stress, rumination and somatic symptoms should be considered multidirectional and multifactorial [26]. Although the a priori power analysis indicated that our sample size is sufficient for this type of analysis, the relatively small sample size of the study may be another limitation. For instance, the significant difference in the correlation of COVID-related rumination and perceived stress between the two groups implies a stronger relationship between these variables in the migraine group, however, no significant interaction was found between headache status and COVID-related rumination. This may be due to the small number of participants per group, therefore further studies are needed to explore these associations on bigger samples. Furthermore, our participants were maximum 50 years old, and the mean age of our sample was 30.76 years, whereas COVID-19 and related difficulties may be more burdensome for the elderly due to their higher risk of mortality and developing severe symptoms [56, 57]. However, other results demonstrated that younger age was associated with more worries about COVID-19, whereas older age was associated with better emotional adaptation and stress reactivity in the current situation [58]. Another limitation is that only healthy adults without severe somatic, neurological or psychological problems were included, although people with such conditions may be more prone to experience severe stress in the current situation [59, 60]. Although beyond the scope of this paper, examining the associations of perceived stress and rumination among at-risk groups would be crucial (for a review of at-risk groups, see Panchal et al. [61]). Other forms of repetitive thinking such as worry, anticipation and health anxiety may also be relevant to the level of perceived stress and would be important to explore in the current situation.

## Conclusions

We did not find any difference in the level of perceived stress among migraineurs and the control group, that may be explained by individual differences in the subjective experience of the COVID-19 situation; some people may feel more relaxed than usual by being able to stay at home, while others may experience more stress and anxiety due to the pandemic. In the complex interplay of stress and migraine, the reaction given to the stressor appears to be more relevant than the stressor itself, highlighting the need for protective factors. In line with this, our results showed that both COVID-related rumination and brooding were associated with higher levels of perceived stress, and these relationships appear to be slightly stronger among migraineurs—however, these can only be inferred from our correlational analyses and need to be interpreted cautiously. These results may imply the increased vulnerability of this patient group in stressful situations like the COVID-19 pandemic, however, the current situation has triggered a mental health crisis among the whole population [4]. This conveys that interventions aiming to reduce depressive and COVID-related rumination and enhance the use of more adaptive coping strategies [62] may contribute to people’s well-being, especially in case of vulnerable groups with stress-related disorders such as migraine patients. Psychoeducation on stress reduction could contribute to healthy adults’ wellbeing and may ease migraineurs’ disease burden by reducing one of the most frequent migraine triggers [63]. For instance, implementing the daily use of electric diaries may help to control the level of perceived stress and related rumination. Interventions aiming to reduce stress and rumination, such as mindfulness-based stress reduction and autogenic training may also be effective [64, 65]. Making telemedicine available for migraineurs could also contribute to reduce their level of stress by offering help safely [66].

## Data Availability

The datasets used and analyzed during the current study are available from the corresponding author on reasonable request.

## Abbreviations

COVID/COVID-19: Coronavirus disease of 2019
HC: Healthy control
RRS: Ruminative Response Scale
PSS/ PSS-4: Perceived Stress Scale
CRS: COVID-related Rumination Scale
PEPQ: Post-event processing questionnaire
MIDAS: The Migraine Disability Assessment
